# Regional Differentiation of Olive Oil of the Koroneiki Olive Cultivar from the Ionian Islands Based on Key Volatile Compounds and Descriptive Data Analysis

**DOI:** 10.3390/foods14234026

**Published:** 2025-11-24

**Authors:** Nikolaos Kopsahelis, Ioannis K. Karabagias, Effimia Eriotou

**Affiliations:** 1 Department of Food Science and Technology, Ionian University, 28100 Argostoli, Greece; eriotou@ionio.gr; 2 Department of Food Science and Technology, School of Agricultural Sciences, University of Patras, G. Seferi 2, 30100 Agrinio, Greece

**Keywords:** Koroneiki cv., olive oil, volatile compounds, HS-SPME/GC-MS, geographical origin, classification, statistics

## Abstract

Given that olive oil produced in the Ionian islands has not been extensively studied, forty-seven olive oil samples of the Koroneiki olive cultivar were collected from Zakynthos, Kerkyra, Kefalonia, and Leukada, which comprise well-known islands of the Ionian Sea. The samples were subjected to analysis of volatile compounds using headspace solid-phase microextraction coupled to gas chromatography–mass spectrometry (HS-SPME/GC-MS). Based on HS-SPME/GC-MS analysis, twenty-five volatile compounds were tentatively identified and semi-quantified using the internal standard method. Volatile compounds included alcohols, aldehydes, benzene derivatives, esters, hydrocarbons, ketones, and terpenoids. The application of descriptive data analysis, such as multivariate analysis of variance (MANOVA), linear discriminant analysis (LDA), and factor analysis (FA), to the semi-quantitative data (μg/L) of the identified volatile compounds resulted in the extraction of key volatile compounds that differentiated olive oil samples of the Koroneiki olive cultivar according to geographical origin. The cross-validation method of LDA showed a prediction rate of 83.0%, whereas the variance explained by FA was approximately 69.06% (69.055%). The key volatile compounds that were associated most with the geographical origin of olive oil samples were (Z)-1,3-pentadiene, dodecane, 2,2,4,6,6-pentamethyl-heptane, 3-ethyl-1,5-octadiene, 2,4-dimethyl-heptane, 4-methyloctane, ethanol, 2-hexanol, and 3-methylbutanal, among others. The study contributes to the regional features of olive oil of the Koroneiki olive cultivar from the Ionian islands based on key volatile compounds and further supports the consecutive research of the international community regarding olive oil authentication.

## 1. Introduction

For ages, nations in the Mediterranean region have used olive oil, a premium vegetable oil. It has grown in favor among consumers in Western Europe, North America, Australia, and other regions, such as Tunisia, because of its exceptional sensory quality, health and nutritional properties (high in monounsaturated oleic acid, polyphenols, squalene), and other bioactive compounds [1,2]. The production of olive oil in the Mediterranean region also plays a very important economic and social role. Spain is the leading producer country, followed by Greece and Italy [3].

Olive oil has been the subject of adulteration or mislabeling because of its high added value [4]. Indeed, nowadays the price of olive oil has greatly increased, given the knowledge of its beneficial health attributes and special application to foods. Concerning its health benefits, research studies have reported a decreased risk of cardiovascular disease, cancer, and other pathophysiological disorders [5]. Acknowledging the issue, the European Union has implemented a set of rules to certify virgin olive oils that meet EU standards known as TSG (Traditional Specialty Guaranteed), PDO (Protected Designation of Origin), and PGI (Protected Geographical Indication). These certifications increase the product’s market value by ensuring that its quality is directly related to its botanical and territorial origin [6].

The differentiation of olive oils is predicated on the identification of various physicochemical and biochemical parameters, such as triglycerides, sterols and fatty acid composition [7,8,9,10], minerals [11], phenolic compounds and pigments [12], volatile compounds [8,13,14], and other micro-constituents, using a plethora of analytical techniques such as gas chromatography (GC), gas chromatography coupled to mass spectrometry (GC/MS), high-performance liquid chromatography (HPLC), high-performance liquid chromatography coupled to mass spectrometry (HPLC/MS), nuclear magnetic resonance (NMR) spectroscopy, isotope ratio mass spectrometry (IRMS), atomic absorption spectroscopy (AAS), inductively coupled plasma spectroscopy coupled to mass spectrometry (ICP/MS), fluorescence spectrometry, near-infrared (NIR) spectroscopy, etc. [15,16]. Among the analytical techniques, solid-phase microextraction coupled to gas chromatography–mass spectrometry (HS-SPME/GC-MS) is a rapid, sensitive, and robust analytical technique that has been routinely used in recent years for the determination of volatile and semi-volatile compounds in numerous foods, including olive oil [1,8,13,17]. The volatile fraction of olive oil consists of carbonyl compounds, alcohols, esters, hydrocarbons, acids, ethers, terpenoids, benzene, and furan derivatives [17,18]. More specifically, the C6 unsaturated and saturated aldehydes, along with the C6 and C5 compounds, comprise the most abundant volatile compounds that are enzymatically generated from polyunsaturated fatty acids via the lipoxygenase (LOX) pathway. These compounds contribute mainly to green odor notes [19]. To effectively study the effect of botanical or geographical origin on the volatile composition of olive oil, descriptive data analysis, also known as chemometrics or statistical analysis, is required. Indeed, descriptive data analysis and predictive informatics are the combination of prescient modeling and informatics connected to food science, healthcare, pharmaceutical, life sciences, and commerce businesses’ data. Descriptive data analysis is the first step in data treatment that empowers analysts, researchers, and decision-makers to aggregate and analyze different sorts of information, recognize designs and patterns within that information, and make more educated choices in an effort to preemptively change future outcomes or predictions (predictive data analysis) regarding the purity, quality, or geographical origin of different olive oils [14,20]. For these mathematical treatments, both supervised (linear discriminant analysis, partial least squares-discriminant analysis, *k*-nearest neighbors, etc.) and unsupervised (principal component analysis, factor analysis, cluster analysis, hierarchical cluster analysis, etc.) statistical techniques are implemented [1,11,13,14,17,20].

Considering the above, the present study aimed to investigate the effect of geographical origin on the volatile composition of olive oil of the Koroneiki olive cultivar, a well-known olive cultivar around the world, from four Ionian islands: Zakynthos, Kerkyra, Kefalonia, and Leukada. In the sampling design of this study, olive oil samples of the Koroneiki olive cultivar from Kefalonia and Leukada were introduced, as we noticed an existing gap in the literature regarding the volatile composition of olive oil from these regions, and limited studies are available [13]. Furthermore, an effort was made to indicate any new volatile markers that could be associated with the geographical origin of olive oil of the Koroneiki olive cultivar grown in four Ionian islands: (i) applying descriptive data analysis such as MANOVA, LDA, and FA; (ii) investigating whether stronger validation could be obtained compared to previous similar studies [13,17]; and (iii) providing new supporting information to the research concerning olive oil geographical origin authentication by indicating the analytical classification results of olive oil samples or potential regional correlations.

## 2. Materials and Methods

### 2.1. Olive Oil Samples

Forty-seven virgin olive oil (VOO) samples (N = 47) of the Koroneiki olive cultivar were analyzed in the present study, harvested during the period October–December 2017 under conventional cultivation, considering that the fruits should have the same degree of ripeness (defined as the time when the fruit begins to change color). Regarding the sampling areas, the collection of samples included as many as possible of the dominant olive growing areas of the Koroneiki cultivar in the Ionian Islands. Therefore, the olive oil samples were collected from four different regions in the Ionian islands: Kefalonia (38°15′54″ N 20°33′09″ E) (12 samples), Kerkyra (39°35′28.60″ N 19°51′50.54″ E) (11 samples), Leukada (38°43′ N 20°39′ E) (7 samples), and Zakynthos (37°48′ N 20°45′ E) (17 samples). Soon after receiving olives (approximately 3 kg/sample), a selection was made to use only healthy olives without defects. Then, olives were crushed to remove the olive pit, followed by grinding in a blender. Afterward, an equal amount of water was added, and the olive mass was kneaded for 45 min at a temperature below 27 °C. The mixture was then centrifuged for 4 min at 3500 rpm. Finally, the olive oil was received, flashed with nitrogen, archived, and placed in dark-colored vials under refrigeration at 4 °C until analyses were performed.

### 2.2. Chemicals and Reagents

The internal standard, 4-methyl-2-pentanone [(CH_3_)_2_CHCH_2_COCH_3_, molecular weight (MW) = 100.16], was purchased from Fluka (Seelze, Germany). The standard mixture of alkanes C_8_–C_20_ (40 mg/L each in *n*-hexane) was purchased from Sigma-Aldrich (Darmstadt, Germany).

### 2.3. Determination of Volatile Compounds in Olive Oil Samples Using HS-SPME/GC-MS

#### 2.3.1. HS-SPME: Sampling, Optimization, and Parametric Conditions

The olive oil samples (4 g) were initially placed in glass vials of 20 mL with screwcaps of polytetrafluoroethylene (PTFE)/silicone septa. Then, a volume of 100 μL of 4-methyl-2-pentanone (internal standard) at an initial concentration of 500 μg/L was added. The vials were sealed and then vortexed for 2 min to homogenize the obtained mixture (olive oil sample + internal standard). Afterward, the vials were maintained in a water bath at 45 °C under continuous stirring at 600 rpm during the extraction procedure. The extraction procedure when the fiber was inserted into the vials was optimized according to our previous study [14], including equilibration time, sampling/exposure time of the fiber, weight of sample, volume of vial, and the temperature of the water bath. The extraction of volatile compounds in the headspace of olive oil samples was performed by using a divinylbenzene/carboxen/polydimethylsiloxane (DVB/CAR/PDMS) fiber (50/30 μm) with a 2 cm length (Supelco, Bellefonte, PA, USA). Before the analysis of olive oil samples, the fiber was cleaned daily using the “clean” program method compatible with the GC-MS procedure that follows.

#### 2.3.2. GC-MS Instrumentation and Conditions of Analysis

The Agilent 7890A gas chromatograph unit coupled to a mass spectrometry detector (Agilent 5975) was used for the analysis of volatile compounds in olive oil samples. The capillary column used was a DB-5MS [cross-linked (5%-Phenyl)-methylpolysiloxane)] capillary column (J & W Scientific, Agilent Technologies, Santa Clara, CA, USA), with dimensions of 60 m × 320 μm i.d., 1 μm film thickness. The carrier gas was helium of high purity (99.999%), at a flow rate of 1.5 mL/min. The optimized conditions of the analysis were the same as those analytically given in our previous study [14]. To avoid any contamination that could cause problems in the reliability of the analysis, blank runs were carried out before and after the analysis of consecutive olive oil samples [17].

### 2.4. Identification of Volatile Compounds and Semi-Quantification

The Wiley 7 NIST mass spectral library of the National Institute of Standards and Technology (NIST 2005) (J. Wiley & Sons Ltd., West Sussex, England) was used for the identification of volatile compounds. Further confirmation of the identified volatile compounds was achieved by the determination of the linear retention time indices (LRIs). More specifically, the mixture of n-alkanes (C_8_–C_20_) (Table 1) was used to calculate the linear retention index values (Kováts indices), following the methodology of the International Union of Pure and Applied Chemistry (IUPAC) [21]. For the statistical evaluation of data derived from the analysis of volatile compounds, only the identified volatile compounds in replicated samples that had a probability higher than 80%, according to the NIST mass spectral library, were considered. Results were expressed as semi-quantitative data (C, μg/L) with reference to the internal standard (IS) 4-methyl-2-pentanone, assuming a response factor equal to 1 [22] following the equation:

C (μg/L) = (Area_Volatile compound_/Area_IS_) × C_IS_ (1)

The internal standard used had a high yield (>95%) and showed no dimerization or co-elution problems.

### 2.5. Descriptive Data Analysis

For the statistical analysis of the results, the semi-quantitative data (μg/L) of volatile compounds were subjected to different multivariate statistical techniques to have more reliable results concerning the differentiation rate of olive oil samples of the Koroneiki olive cultivar according to geographical origin. At first, the average values of the analyzed samples were compared using MANOVA to determine which volatile compounds showed significant differences (*p* < 0.05) in their composition among olive oil samples of different geographical origin (Zakynthos, Kerkyra, Kefalonia, and Leukada). In principle, MANOVA creates linear combinations of all the dependent variables in the model (i.e., volatile compounds), that maximize the differences in the average values between the level groups of the independent variables (i.e., geographical origin). MANOVA uses the Wilks’ Lambda and Pillai’s Trace tests to indicate the main effects and interactions of the independent variables at the multidimensional level [14]. Normally, MANOVA is followed by LDA, which is a supervised statistical technique. LDA indicates the linear combinations of the statistically significant (*p* < 0.05) parameters of interest (i.e., volatile compounds indicated during MANOVA) that separate two or more groups of objects (i.e., geographical origin of olive oil samples). The prediction ability of the LDA models was estimated based primarily on the cross-validation method, also known as leave-one-out cross-validation, during which the model is explicitly trained on the entire dataset except for one data point (sample), which is used for testing. This process is repeated for each sample in the dataset. Therefore, all samples are used for training, resulting in low bias and providing more accurate results compared to the original method of LDA [14]. For the LDA, the geographical origin of olive oil samples was considered as the factor variable (grouping variable), while the semi-quantitative data of the volatile compounds as the independent variables. To test for the homogeneity of variances for normally distributed samples, Bartlett’s test of sphericity was considered [23].

Finally, a dimension reduction technique such as FA (an unsupervised statistical technique) was used to describe the variance that exists between an initial number of measured (called as obvious) and associated variables, and a smaller number of non-obvious variables, called factors. The purpose of FA is to summarize the relationships between the initial and the factor variables comprehensively and accurately by providing percentages of variance (% variance) associated with those factors. During FA, the Kaiser-Meyer-Olkin (KMO) index criterion that assesses the sample adequacy (it should be >0.50), and Bartlett’s Test of Sphericity (*p*-value should be <0.05) that assesses whether the correlations between the variables allow the effective implementation of FA must be considered. The extraction method during FA was principal component analysis (PCA), with Varimax rotation, an orthogonal rotation where all factors remain uncorrelated with one another, and Kaiser Normalization that provides stability features during the statistical treatment of samples [17]. Predictive informatics was performed using the Statistical Package for the Social Sciences statistics software (SPSS) version 26.0 (IBM Inc., Armonk, NY, USA, 2019).

## 3. Results

### 3.1. Volatile Compounds of Olive Oil of the Koroneiki Olive Cultivar from the Ionian Islands

The semi-quantitative data of the volatile compounds that were tentatively identified in olive oil samples of the Koroneiki olive cultivar according to geographical origin are shown in Table 2.

Significant (*p* < 0.05) differences in the volatile composition (μg/L) of olive oil samples of the Koroneiki olive cultivar according to geographical origin were observed for the majority of the identified volatile compounds. The most abundant volatile compounds were aldehydes, followed by hydrocarbons and alcohols. However, considerable amounts of the volatile compounds belonging to other classes were determined (Table 2). The order for the average composition of alcohols according to geographical origin was: Kefalonia > Zakynthos > Leukada > Kerkyra. In the case of aldehydes, the respective order in their average composition was: Kerkyra > Leukada > Zakynthos > Kefalonia. The respective order in the average composition of benzene derivatives was Leukada > Zakynthos > Kerkyra > Kefalonia. Similarly, esters showed the following order in their average composition: Kefalonia > Zakynthos > Leukada > Kerkyra. Hydrocarbons, in turn, had the following order in their average composition: Zakynthos > Kerkyra > Leukada > Kefalonia. For ketones, the respective order in their average composition was Zakynthos > Kerkyra > Kefalonia. Finally, terpenoids were identified only in Kerkyra. Four alcohols were identified among the olive oil samples studied. Ethanol was identified in olive oil samples from Kefalonia and Leukada, recording the highest amount in olive oil samples from Kefalonia. In addition, 1-propanol was identified only in olive oil samples from Kefalonia. 2-Hexanol was identified only in olive oil samples from Kerkyra. Concerning the other alcohols, (Z)-3-hexen-1-ol recorded the highest amount in olive oil samples from Kefalonia, followed by those of Zakynthos (Table 2).

**Figure 1:**
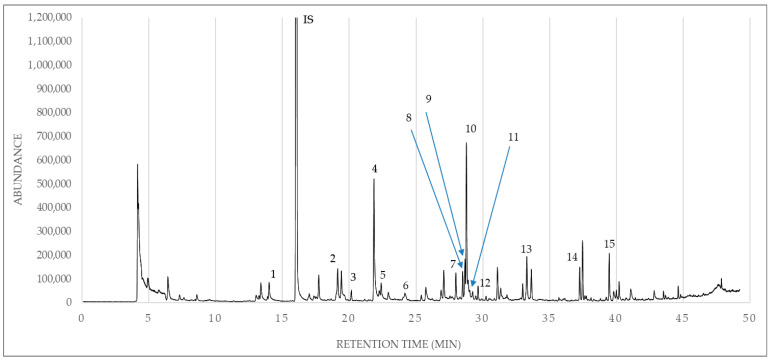
A representative chromatogram of olive oil of the Koroneiki olive cultivar from Zakynthos. IS: internal standard. 1: pentanal. 2: hexanal. 3: 2,4-dimethylheptane. 4: (Z)-3-hexen-1-ol. 5: 4-methyloctane. 6: heptanal. 7: 6-methyl-5-hepten-2-one. 8: 2,2,4,6,6-pentamethylheptane. 9: decane. 10: (Z)-3-hexen-1-ol, acetate. 11: 2,5-dimethylnonane. 12: 4-methyldecane. 13: nonanal. 14: dodecane. 15: 1,3-bis (1,1-dimethylethyl)benzene.

**Figure 2:**
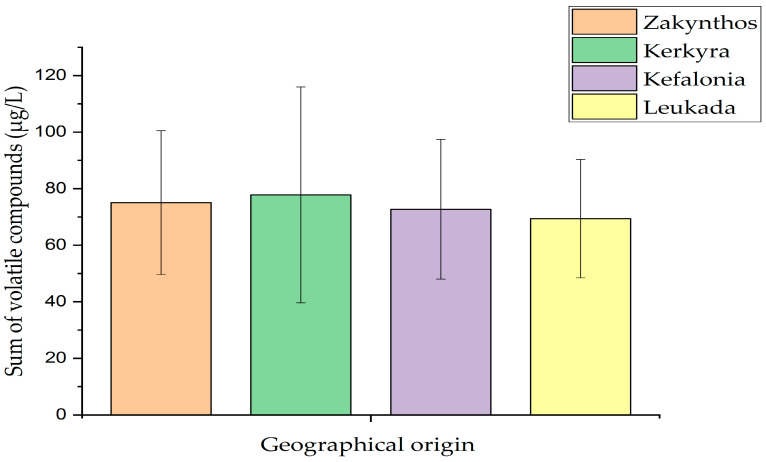
Sum of volatile compounds classes according to the geographical origin of olive oil of the Koroneiki olive cultivar from the Ionian islands. Error bars indicate statistically significant differences at the confidence level *p* < 0.05.

Regarding aldehydes, 3-methylbutanal was identified only in olive oil samples from Leukada, whereas pentanal recorded the highest amount in olive oil samples from Kerkyra. Hexanal recorded the highest amount in olive oil samples from Kefalonia, followed by those of Zakynthos. What is remarkable is that (E)-2-hexenal was the most abundant aldehyde in amount and recorded the highest value in olive oil samples from Kerkyra, followed by those of Leukada. Of the other aldehydes, heptanal was identified only in olive oil samples from Zakynthos. Finally, nonanal recorded the highest amount in olive oil samples from Zakynthos, followed by those of Leukada, Kerkyra, and Kefalonia. Of the benzene derivatives, toluene was identified in the highest amount in olive oil samples from Leukada, followed by those of Kerkyra. In parallel, 1,3-bis(1,1-dimethylethyl)-benzene was identified in all olive oil samples, recording the highest amount in olive oil samples from Leukada, followed by those of Zakynthos, Kefalonia, and Kerkyra (Table 2). Considerable amounts of hydrocarbons were recorded, and some special findings were observed. In particular, (Z)-1,3-pentadiene, 1,4-pentadiene, and 3-ethyl-1,5-octadiene were identified only in olive oil samples from Kerkyra, while 4-methyloctane was identified only in olive oil samples from Zakynthos. Of the remaining hydrocarbons, 2,4-dimethylheptane was identified in the highest amount in olive oil samples from Zakynthos, followed by those of Kerkyra and Kefalonia. The compound 2,2,4,6,6-pentamethylheptane recorded the highest amount in olive oil samples from Kefalonia, followed by those of Leukada, Zakynthos, and Kerkyra. On the contrary, decane recorded the highest amount (μg/L) in olive oil samples from Zakynthos, followed by those of Kerkyra, Kefalonia, and Leukada. 2,5-Dimethylnonane recorded the highest amount in olive oil samples from Kerkyra, followed by those of Zakynthos. Furthermore, 4-methyldecane recorded the highest amount in olive oil samples from Kerkyra, followed by those of Leukada and Zakynthos. Finally, dodecane recorded the highest amount in olive oil samples from Zakynthos, followed by those of Kerkyra, Leukada, and Kefalonia (Table 2). Of the ketones, only 6-methyl-5-hepten-2-one was identified in olive oil samples from Zakynthos, Kerkyra, and Kefalonia. The highest amount of this compound was recorded in olive oil samples from Zakynthos, followed by those of Kerkyra and Kefalonia. (Z)-3-hexen-1-ol, acetate was the only esterified compound that was identified in olive oil samples, recording the highest amount in olive oil samples from Kefalonia, followed by those of Zakynthos, Leukada, and Kerkyra. Finally, terpenoids showed a relatively low contribution to the overall volatile profile of olive oil samples of the Koroneiki olive cultivar from the Ionian islands, given that dl-limonene was the only identified terpenoid compound in olive oil samples from Kerkyra. A representative gas chromatogram of an olive oil sample from Zakynthos is shown in Figure 1. Notably, Figure 2 shows the sum of volatile compound classes according to the geographical origin of olive oil of the Koroneiki olive cultivar from the Ionian islands, which followed the order Kerkyra (77.76 ± 38.18 μg/L) > Zakynthos (75.07 ± 25.44 μg/L) > Kefalonia (72.72 ± 24.69 μg/L) > Leukada (69.38 ± 20.95 μg/L).

### 3.2. Regional Differentiation of Olive Oil Samples of the Koroneiki Olive Cultivar from the Ionian Islands

#### 3.2.1. MANOVA

MANOVA analysis revealed that the geographical origin of olive oil samples from the Koroneiki olive cultivar influenced their volatile composition (μg/L). More specifically, Pillai’s Trace = 2.671 (F = 6.812, df = 75, *p* = 0.000), and Wilks’ Lambda = 0.001 (F = 7.667, df = 75, *p* = 0.000), approved this significant impact. In addition, Bartlett’s test of sphericity (*X*^2^ = 2869.90, df = 324, *p* < 0.001) indicated the differences in the variances across the normally distributed samples, rejecting the null hypothesis that all variances are equal. In total, 21 volatile compounds showed significant differences in their composition (μg/L) with respect to the geographical origin of olive oil samples (Table 2).

#### 3.2.2. LDA

Following MANOVA, LDA classified olive oil samples according to geographical origin. Table 3 and Table 4 show all the analytical data of the LDA. In total, three discriminant functions were formed, which explained 100% of the total variance.

**Figure 3:**
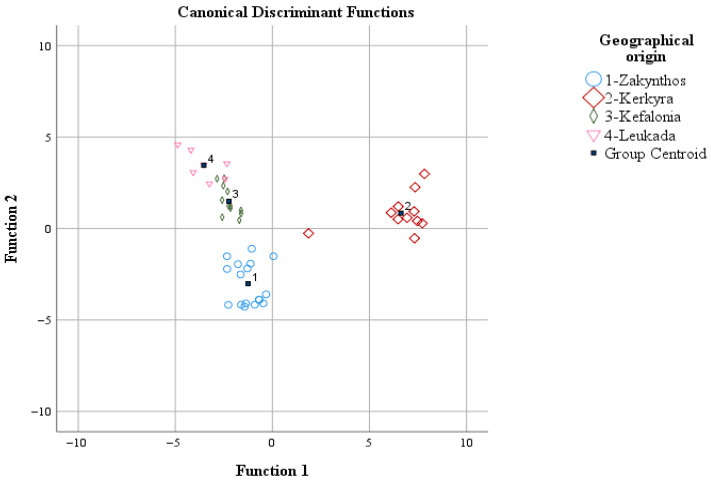
Geographical origin differentiation of olive oil of the Koroneiki olive cultivar from the Ionian islands based on volatile compounds and LDA.

The most encouraging results were obtained for the olive oil samples from Zakynthos, where out of 17 initial samples, 16 were correctly allocated to Zakynthos (correct classification rate of 94.1%), while 1 sample was allocated to Kefalonia. Accordingly, for Kerkyra, out of the 11 initial samples, 8 were correctly classified in Kerkyra (correct classification rate of 72.7%), while 2 samples were allocated to Zakynthos and 1 to Leukada. Additionally, for the olive oil samples from Kefalonia, out of the 12 initial samples, 10 were correctly allocated to Kefalonia (correct classification rate of 83.3%), while 2 samples were allocated to Zakynthos. Finally, for the olive oil samples from Leukada out of the 7 initial samples, 5 were correctly allocated to Leukada (correct classification rate of 71.4%), while 2 were allocated to Kefalonia. The overall correct classification rate was 97.9% for the original and 83% for the cross-validation method. Between these values, the latter is considered very satisfactory given that samples of olive oil of the same cultivar were studied in terms of geographical origin differentiation (Figure 3). The group centroid values of the discriminant functions (coordinates) of LDA were: (−1.251, −3.017), (6.624, 0.841), (−2.240, 1.489), and (−3.531, 3.454) for Zakynthos. Kerkyra, Kefalonia, and Leukada, respectively.

The volatile compounds that contributed the most to the differentiation of olive oil samples of the Koroneiki olive cultivar from the Ionian islands were those pooled with the highest absolute correlation value within the discriminant functions. These volatile compounds are indicated in bold lettering in Table 5. Therefore, these volatile compounds are most strongly related to the geographical origin of olive oil samples of the Koroneiki olive cultivar from the Ionian islands.

#### 3.2.3. FA

FA showed that the 21 statistically significant (*p* < 0.05) volatile compounds robustly describe the variability in the multidimensional space. The Keiser-Meyer-Olkin (KMO) index was 0.601, while Bartlett’s Test of Sphericity index had the values Chi-square = 529.816, df = 210, *p* < 0.001, indicating the existence of correlations between the variables (volatile compounds), thus allowing the application of factor analysis. The main volatile compounds that showed the highest correlation (components/factors) are given in bold letters in Table 6.

**Figure 4:**
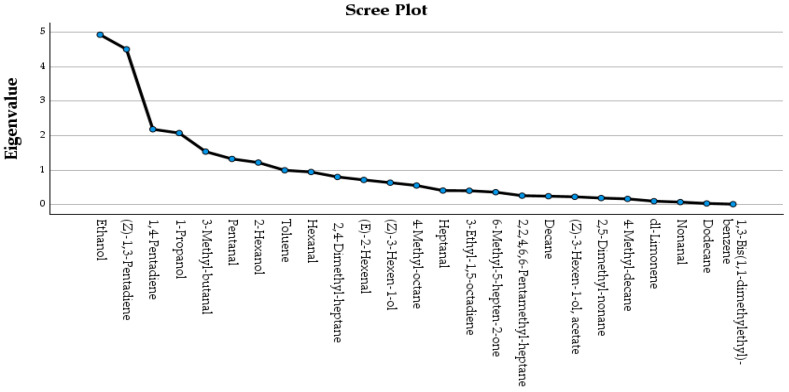
Scree plot (eigenvalue) of the identified volatile compounds in olive oil of the Koroneiki olive cultivar from the Ionian islands during FA.

Based on the first 6 principal components (PCs), the variance explained was 69.055%, a value considered satisfactory. The volatile compounds for which the correlation value in the rotated component matrix of the multidimensional space was the largest were: dodecane (PC1, 16.130% of the total variance), 1,4-pentadiene (PC2, 15.530% of the total variance), 2,4,6,6-pentamethylheptane (PC3, 9.959% of the total variance), 3-methylbutanal (PC4, 9.760% of the total variance), (Z)-1,3-pentadiene (PC5, 9.176% of the total variance and 1-propanol (PC6, 8.499% of the total variance). The scree plot (eigenvalue) of the identified volatile compounds during FA is shown in Figure 4.

## 4. Discussion

The volatile composition of olive oil is influenced by several factors, including cultivar, geographic region, edaphoclimatic and agronomic conditions (such as fertilization and irrigation), natural product maturity, and handling methods (such as temperature and malaxation time) [10,13,14]. Normally, the C5 and/or C6 compounds are the major constituents of the olive oil aroma fraction and, in particular, linear unsaturated and saturated aldehydes with 6 carbon atoms. The biosynthesis of these substances is due to the olive tissues being broken after the lipoxygenase (LOX) pathway, a cascade of enzymes acting on polyunsaturated fatty acids [24].

It is important to note that the volatile compounds identified in this study (Table 2) align with previous research on the cultivar or geographic origin authentication of olive oil from various countries [13,14,25,26]. The key volatiles that contribute to olive oil aroma include hexanal, which has a scent associated with green apple and cut grass, trans-2-hexenal (or (E)-2-hexenal), which is related to sharp almond aroma, and other green, fruity, sharp, biting, and astringent notes, as well as 1-hexanol, which has a smell similar to tomato and other fruity, delicate, fragrant, and alcoholic or even pungent odors [27]. Additionally, the role of aldehydes in differentiating olive oil by cultivar or geographic origin has been thoroughly documented in previous studies [13,14,17,25,26], including samples of the Koroneiki olive cultivar from various parts of the world [28,29]. Past research has linked 1-hexanol to olive oil from the Koroneiki olive cultivar and specific regions, such as Greek areas like Messinia, Lakonia, Irakleio, Etoloakarnania, and Zakynthos [29,30]. This volatile compound enhances the oil’s strong, grassy, and fruity aroma notes [31].

Furthermore, we should also consider how the region (weather and soil), as well as agricultural and technical factors, can affect the volatile makeup of the olive oil varieties being studied. Indeed, in our case, we identified an isomer of 1-hexanol, that of 2-hexanol, which has never been identified previously in olive oil of the Koroneiki olive cultivar [29,30]. Ethanol can also give olive oil a fermented, overripe fruit, and strong smell [14]. In previous studies, ethanol was reported to contribute to the aroma of olive oil of the Koroneiki olive cultivar from Zakynthos and Peloponnese [8,24,28]. However, 1-propanol was not reported previously to contribute to the aroma of olive oil of the Koroneiki olive cultivar from Messinia, Lakonia, Irakleio, and Etoloakarnania [29].

Considering the length of the carbon chain, hydrocarbons can produce a fragrant, pleasant, apple-like, and greasy smell in olive oil [32]. Hydrocarbons can also originate from the LOX pathway [14]. Another important aspect to consider is that the hydrocarbons identified in the present study (except for decane and (Z)-1,3-pentadiene) were not previously reported to contribute to the scent of olive oil from the Koroneiki olive cultivar [30].

Furthermore, 6-methyl-5-hepten-2-one has been reported to have a special role in the scent of olive oil from different regions in Greece, including, among others [29], the Ionian islands [14,17,30], whereas it was not previously reported to contribute to the aroma of olive oil samples from Italy [31] or Morocco [32].

The benzene derivative, 1,3-bis(1,1-dimethylethyl)benzene, was not previously reported to contribute to the aroma of olive oil of the Koroneiki olive cultivar from Messinia, Lakonia, Etoloakarnania, and Irakleio [29]. The same holds for olive oil of the Koroneiki olive cultivar from Morocco (Picholine marocaine cultivar) [32] and the Alperujo olive cultivar from Italy [33]. Toluene and 1,3-bis(1,1-dimethylethyl)benzene may lead to a severe and hot olive oil flavor [14]. 1,3-Bis(1,1-dimethylethyl)benzene may be related to the olive oil of the Koroneiki olive cultivar grown in the Ionian Islands; however, further research is required.

The volatile compound (Z)-3-hexen-1-ol, acetate (or Z-3-hexenyl-acetate) was previously reported to contribute to the aroma of olive oil samples of the Koroneiki olive cultivar from Zakynthos [24], in agreement with the results of the present study. This ester may also originate from the LOX pathway and create fruity, sweet, and enjoyable-smelling hints of olive oil [31]. Lastly, the volatile compound dL-limonene can differ in its content based on the type of olive cultivar and geographical origin [14,17,24,29].

Concerning the differentiation of olive oil of the Koroneiki olive cultivar, according to geographical origin, the classification results obtained for the studied olive oil samples using LDA (correct classification rate of 83% for the cross-validation method) are in good agreement with those reported in previous studies [8,13,24,29] using LDA, which ranged between 79.7 and 87.2%. Similarly, the application of dimension reduction techniques, such as PCA, proved to be efficient for the characterization of the volatile composition of olive oil samples of different olive cultivars from Tunisia [34], providing an overall variance explained equal to 62.70%. Similarly, the use of FA in the present study provided the key volatile compounds that were associated mostly with the geographical origin of olive oil samples of the Koroneiki olive cultivar (total variance explained was slightly over 69%), in agreement with previous studies in the literature concerning olive oil obtained from olive cultivars grown in the Ionian islands [14,17].

## 5. Conclusions, Limitations, and Future Perspectives

The analysis of the volatile components of olive oil samples of the Koroneiki olive cultivar from different geographical regions in the Ionian islands proved to be an important tool for differentiating the geographical zone in which the olive oil was produced, with the help of descriptive data analysis, highlighting some key volatile compounds and volatile compounds that had not been previously identified in olive oil of the Koroneiki olive cultivar such as 2-hexanol, 1-propanol, 1,3-bis(1,1-dimethylethyl)benzene, and numerous hydrocarbons. More specifically, LDA indicated the volatile compounds that showed the highest absolute correlation with the specific regions, including, among others, (Z)-1,3-pentadiene, dodecane, 2,2,4,6,6-pentamethyl-heptane, 3-ethyl-1,5-octadiene, 2,4-dimethyl-heptane, 4-methyloctane, ethanol, 2-hexanol, and 3-methylbutanal, providing an overall classification rate of 83%. In addition, FA complemented LDA and further confirmed the obtained results by providing a correlation of the key volatile compounds with the geographical origin of olive oil of the Koroneiki olive cultivar. Considering that the differentiation/identification of the geographical origin of products is a difficult task, the present results are considered very satisfactory, even preliminary in nature. Beyond the characterization of the aroma of the olive oil of the Koroneiki olive cultivar of different geographical origin, the research contributes to the authenticity of the olive oil from Ionian islands, providing knowledge and financial and other benefits for all involved parties and giving incentives for its further accreditation as a product of a protected geographical zone, considering the high market value of olive oil nowadays. Finally, the collection of a higher number of olive oil samples through consecutive harvesting years from these regions, the analysis of independent datasets to proceed to predictive informatics, and the implementation of additional analytical techniques such as high-pressure liquid chromatography coupled to tandem mass spectrometry (LC-MS/MS) will further strengthen the present results based on the determination of non-volatile compounds (i.e., phenolic compounds) that could complementarily serve as regional markers of olive oil of the Koroneiki olive cultivar grown in the Ionian islands.

## Figures and Tables

**Table 1:** Retention time of the standard mixture of alkanes.

Standard Alkanes	Retention Time (min)
C_8_	16.20
C_9_	20.99
C_10_	25.66
C_11_	29.84
C_12_	33.96
C_13_	38.01
C_14_	41.00
C_15_	43.55
C_16_	45.50
C_17_	47.30
C_18_	49.10
C_19_	51.05
C_20_	53.52

**Table 2:** Volatile compounds tentatively identified in olive oil of the Koroneiki olive cultivar from the Ionian islands.

Retention Time (RT, min)	Volatile Compounds (μg/L)	LRI ^a^	Zakynthos (Avg ± SD)	Kerkyra (Avg ± SD)	Kefalonia (Avg ± SD)	Leukada (Avg ± SD)	Wilks’ Lambda	F	*p*
	*Alcohols*								
5.70	Ethanol	<800	Nd	Nd	5.09 ± 3.27	2.48 ± 2.03	0.390	22.459	<0.001
8.13	1-Propanol	<800	Nd	Nd	7.11 ± 13.40	Nd	0.814	3.278	0.030
16.99	2-Hexanol	816	Nd	0.89 ± 0.95	Nd	Nd	0.578	10.480	<0.001
21.87	(Z)-3-Hexen-1-ol	920	11.38 ± 12.54	Nd	13.80 ± 11.62	Nd	0.696	6.246	0.001
	Sum of alcohols (Avg)		11.38	0.89	26.00	2.48			
	*Aldehydes*								
12.09	3-Methyl butanal	<800	Nd	Nd	Nd	0.14 ± 0.13	0.481	15.451	<0.001
14.00	Pentanal	<800	2.43 ± 1.82	2.89 ± 2.40	2.67 ± 1.99	2.61 ± 2.27	0.992	0.111	0.953
19.15	Hexanal	862	8.07 ± 2.68	7.66 ± 3.09	8.74 ± 3.41	7.41 ± 4.86	0.979	0.305	0.822
21.86	(E)-2-Hexenal	919	Nd	29.48 ± 40.82	Nd	16.64 ± 23.13	0.730	5.313	0.003
24.16	Heptanal	968	1.54 ± 1.54	Nd	Nd	Nd	0.597	9.682	<0.001
33.30	Nonanal	1184	9.48 ± 5.02	5.15 ± 3.79	4.19 ± 5.08	8.02 ± 6.85	0.820	3.141	0.035
	Sum of aldehydes (Avg)		21.52	45.18	15.60	34.82			
	*Benzene derivatives*								
17.71	Toluene	831	Nd	1.17 ± 1.06	Nd	2.06 ± 2.32	0.600	9.950	<0.001
39.47	1,3-Bis(1,1-dimethylethyl)benzene	1349	4.84 ± 1.35	3.50 ± 2.18	4.17 ± 3.06	5.73 ± 2.46	0.897	1.645	0.193
	Sum of benzene derivatives (Avg)		4.84	4.67	4.17	7.79			
	*Esters*								
28.82	(Z)-3-Hexen-1-ol, acetate	1076	8.03 ± 6.97	6.12 ± 6.78	11.02 ± 9.10	7.63 ± 7.93	0.946	0.826	0.487
	Sum of esters and derivatives (Avg)		8.03	6.12	11.02	7.63			
	*Hydrocarbons*								
7.28	(Z), 1,3-Pentadiene	<800	Nd	0.73 ± 0.81	Nd	Nd	0.592	9.864	<0.001
7.30	1,4-Pentadiene	<800	Nd	0.79 ± 0.92	Nd	Nd	0.613	9.053	<0.001
20.15	2,4-Dimethylheptane	882	2.21 ± 0.98	1.88 ± 0.64	1.05 ± 0.99	Nd	0.518	13.361	<0.001
22.24	4-Methyloctane	927	1.45 ± 1.28	Nd	Nd	Nd	0.537	12.372	<0.001
26.21	3-Ethyl-1,5-octadiene	1013	Nd	0.58 ± 0.56	Nd	Nd	0.527	12.887	<0.001
28.49	2,2,4,6,6-Pentamethylheptane	1068	5.06 ± 1.31	2.39 ± 2.84	7.88 ± 3.04	7.71 ± 2.56	0.541	12.168	<0.001
28.67	Decane	1072	6.89 ± 1.24	6.69 ± 1.76	4.53 ± 1.36	4.43 ± 1.13	0.579	10.443	<0.001
29.46	2,5-dimethylnonane	1091	0.68 ± 0.69	0.96 ± 0.90	Nd	Nd	0.675	6.916	0.001
29.65	4-Methyl decane	1095	1.35 ± 1.49	1.97 ± 1.30	Nd	1.59 ± 0.92	0.695	6.289	0.001
37.30	Dodecane	1283	5.17 ± 1.43	3.85 ± 2.59	1.87 ± 1.42	2.93 ± 0.91	0.610	9.158	<0.001
	Sum of hydrocarbons (Avg)		22.81	19.84	15.33	16.66			
	*Ketones*								
28.01	6-Methyl 5-hepten-2-one	1056	6.49 ± 7.67	0.65 ± 0.72	0.60 ± 0.92	Nd	0.708	5.898	0.002
	Sum of ketones (Avg)		6.49	0.65	0.60	Nd			
	*Terpenoids*								
30.46	1-Methyl-4-(prop-1-en-2-yl)cyclohex-1-ene (dl-Limonene)	1115	Nd	0.41 ± 0.46	Nd	Nd	0.598	9.627	<0.001
	Sum of terpenoids (Avg)		Nd	0.41	Nd	Nd			

Nd: not determined. ^a^. Experimental retention time indices according to the Kováts equation and by using a standard mixture of linear alkanes (C_8_–C_20_). Avg ± SD: Average ± standard deviation. MANOVA in the comparison of the average values at the significance level *p* < 0.05. F: Value of the F-function; *p*: Probability. The variables with a higher F-value contribute most to the classification process.

**Table 3:** Eigenvalues, total variance (%), cumulative variance (%), and canonical correlation of the discriminant functions based on LDA.

Discriminant Functions				
Function	Eigenvalue	% of Variance	Cumulative %	Canonical Correlation
1	15.272 ^a^	60.2	60.2	0.969
2	6.341 ^a^	25.0	85.2	0.929
3	3.750 ^a^	14.8	100	0.889

^a^. First 3 canonical discriminant functions were used in the analysis.

**Table 4:** Qualitative and quantitative characteristics of the discriminant functions based on LDA.

Discriminant Functions				
Test of Function(s)	Wilks’ Lambda	Chi-square	df	Sig. (*p*)
1 through 2	0.002	212.427	63	0.000
2 through 3	0.029	118.980	40	0.000
3	0.211	52.200	19	0.000

Sig: level of significance (*p*).

**Table 5:** Key volatile compounds of olive oil of the Koroneiki olive cultivar from the Ionian islands based on LDA.

Volatile Compounds	Discriminant Function		
	1	2	3
3-Ethyl-1,5-octadiene	0.238 *	0.073	0.028
2-Hexanol	0.214 *	0.066	0.025
2,2,4,6,6-Pentamethylheptane	−0.212 *	0.137	−0.106
(Z)-1,3-Pentadiene	0.208 *	0.064	0.025
dl-Limonene	0.205 *	0.063	0.024
1,4-Pentadiene	0.199 *	0.061	0.024
(Ε)-2-Hexenal	0.123 *	0.115	0.122
4-Methyloctane	−0.060	−0.348 *	0.104
2,4-Dimethylheptane	0.112	−0.337 *	−0.076
Heptanal	−0.053	−0.308 *	0.092
Decane	0.120	−0.271 *	0.106
Dodecane	0.042	−0.267 *	0.206
6-Methyl-5-hepten-2-one	−0.034	−0.246 *	0.054
2,5-Dimethylnonane	0.140	−0.156 *	0.084
Ethanol	−0.166	0.304	−0.386 *
3-Methylbutanal	−0.105	0.247	0.373 *
Toluene	0.052	0.221	0.290 *
4-Methyldecane	0.099	−0.040	0.272 *
(Z)-3-Hexen-1-ol	−0.097	−0.128	−0.224 *
1-Propanol	−0.043	0.069	−0.213 *
Nonanal	−0.041	−0.122	0.162 *

* higher correlation (absolute value).

**Table 6:** Key volatile compounds (components) associated with the geographical origin of olive oil of the Koroneiki olive cultivar from the Ionian islands.

Volatile Compounds	Components					
	1	2	3	4	5	6
Dodecane	0.783 *	−0.129	0.263		0.302	0.233
Nonanal	0.726					
Decane	0.657	0.103	0.170	0.284	0.395	0.319
Heptanal	0.595			0.254	−0.372	0.190
2,4-Dimethylheptane	0.558		0.374	0.470	0.122	
dl-Limonene	−0.517	0.411	0.333	0.159		0.285
4-Methyloctane	0.489	−0.175	0.120	0.206	−0.317	0.314
1,4-Pentadiene	−0.109	0.936 *	0.150			
(E)-2-Hexenal		0.774		−0.242		
3-Ethyl-1,5-octadiene	−0.239	0.758	0.212	0.148	0.178	0.150
2-Hexanol	0.163	0.671			0.543	
2,2,4,6,6-Pentamethylheptane		−0.400	−0.745 *	−0.118		
(Z)-3-Hexen-1-ol	0.223	−0.178	−0.593	0.439	−0.220	0.128
2,5-Dimethylnonane	0.390	0.161	0.515	0.223	0.292	0.185
6-Methyl-5-hepten-2-one	0.278	−0.306	0.450	0.184	−0.208	0.184
4-Methyldecane	0.279	0.156	0.359	−0.238	0.293	0.353
3-Methylbutanal		−0.133		−0.841 *		
Toluene	−0.169	0.348		−0.675	0.154	
(Z)-1,3-Pentadiene			0.136		0.883 *	0.102
1-Propanol				0.114		−0.884 *
Ethanol	−0.382	−0.199	−0.342			−0.605

* higher correlation (absolute value).

## Data Availability

The original contributions presented in this study are included in the article. Further inquiries can be directed to the corresponding author(s).
